# Associations between Body Mass Index and Visual Impairment of School Students in Central China

**DOI:** 10.3390/ijerph13101024

**Published:** 2016-10-18

**Authors:** Fen Yang, Chongming Yang, Yuzhong Liu, Shuzhen Peng, Bei Liu, Xudong Gao, Xiaodong Tan

**Affiliations:** 1 Department of Epidemiology and Biostatistics, School of Health Sciences, Wuhan University, 115# Donghu Road, Wuhan 430071, China; 2014103050005@whu.edu.cn (F.Y.); 2013203050013@whu.edu.cn (Y.L.); 2013103050008@whu.edu.cn (B.L.); 2014103050004@whu.edu.cn (X.G.); 2 College of Nursing, Hubei University of Chinese Medicine, 1# Huangjiahu West Road, Wuhan 430061, China; 3 Research Support Center, Brigham Young University, Provo, UT 84602, USA; chongming_yang@byu.edu; 4 Teenagers Vision Prevention and Control Center, Huangpi District People’s Hospital, 259# Baixiu Street, Wuhan 4300300, China; yf_20062007@126.com

**Keywords:** visual impairment, obesity, Body Mass Index

## Abstract

Body Mass Index (BMI) is a risk indicator for some eye diseases. However, the association between BMI and Visual Impairment (VI) was not quite certain in Chinese students. Our aim was to assess the relationship between BMI and VI with a cross-sectional study. A total of 3771 students aged 6–21 years, including 729 with VI, were sampled from 24 schools in Huangpi District of central China to participate in the study. A multistage stratified cluster random sampling was adopted. Each of the students answered a questionnaire and had physical and eye examinations. The association between BMI and VI was examined with logistic regression and threshold effect analysis. The prevalence of VI was 19.33% (729/3771). Compared to normal and underweight, overweight/obese students showed a stronger relation with VI in age- and sex-adjusted (Odds Ratio (OR) = 16.16, 95% Confidence Interval (CI): 12.37–21.09, *p* < 0.001) and multivariable models (OR = 8.32, 95% CI: 6.13–11.30, *p* < 0.001). There was a nonlinear dose–response relation between levels of BMI and the prevalence of VI (*p* < 0.001). A high level of BMI (≥19.81 kg/m^2^) was associated with a higher VI prevalence (adjusted OR = 1.20, 95% CI: 1.15–1.25, *p* < 0.001). In conclusion, the study demonstrated BMI levels were significantly associated with the prevalence of VI.

## 1. Introduction

There were nearly 285 million people with Visual Impairment (VI) worldwide in 2010 [1]. VI in school-age students is a major public health problem [2,3,4,5]. Good visual acuity (VA) is critical to students’ well-being and VI has many negative effects on all domains of the development of individuals [6,7,8,9,10]. The World Health Organization (WHO) has identified VI as one of the five priority areas in VISION 2020, especially in school-age children of developing countries [11].

Overweight and obesity are currently important health problems affecting school-age students. Overweight children are more likely to become overweight adults and have greater risk of obesity in adulthood than normal weight children [12,13,14,15,16,17]. Obesity has been demonstrated to be negatively associated with VA and other eye diseases among older adults [18]. Body Mass Index (BMI) is an easy measure and useful tool for diagnosing obesity. A recent study found there was a highly significant positive correlation between BMI and VI [19]. Some studies also found that children with VI had higher weights and higher BMIs [20,21,22]. However, the level and the mechanism of this association were not certain in school students. In severe cases, obesity may lead to other diseases that further cause eye diseases [23]. In school students with VI, the association has been attributed to unhealthy lifestyles, such as overuse of eyes (e.g., excessive TV watching and computer use, or doing homework for a long time), insufficient outdoor physical activities, etc. [24,25].

Some researchers have examined this BMI–VI association in developed coastal metropolises or in regions near the national capital [26,27,28], which might not reflect economically less developed areas of China. Wuhan is the largest city in central China with nearly 953 primary and middle schools with 750,000 students (2014 Census). Students enjoy less educational resources (e.g., exercise facilities, extracurricular activities, proportion of students allowed to enter colleges) and experience a greater pressure of learning than those in developed coastal areas, including Beijing or Guangzhou [24,25]. So this study had a representative sample of Central China. Our aim was to explore the connection between BMI and the risk of VI with a nonlinear dose–response analysis. The result of this study should assist relevant agencies in developing appropriate policies to prevent and control VI in school students.

## 2. Methods

### 2.1. Sample

This cross-sectional study was conducted to investigate the prevalence and risk factors of VI in schoolchildren aged 6–21 years living in Huangpi District, one of the 13 administrative districts in Wuhan City. It is located in the east part of Hubei Province at 28 km from the provincial capital Wuhan. Wuhan is an economic and cultural center of Central China located at the Yangtze River. The population of Huangpi district was about 1.13 million in the 2013 census, occupying 2261 km^2^. There are 96 elementary, 27 middle, and 9 high public schools in the district.

The Wuhan Municipal Education Commission and Huangpi Bureau of Education sent relevant information and consent forms to the selected school officials, parents, and students for cooperation between March and April of 2016. Each student and their parents were informed of the study by the head teacher before the survey and examination. The Huangpi Center for Disease Control and Prevention (CDC) got in touch with each selected schools’ authority to explain the purpose and procedures of the project. The CDC gained a list of all the primary and middle school students, including name, age, class, and sex as the sampling frame.

A total of 4250 school-age students were sampled from March to June 2016. The sampling was performed using a multi-stage random cluster approach. Firstly, four streets were randomly selected from all 16 streets in the rural and urban regions of Huangpi District. Secondly, six schools were randomly selected from each of the four selected streets. Finally, two classes of grades 1 to 12 of the selected schools were sampled. Those students who were absent, refused to participate, or did not complete the examination were excluded. Everyone who agreed to attend the study gave his or her written consent to participate in the study.

Students with neuropsychiatric diseases and congenital visual impairments, such as cerebral visual impairments, were excluded from the analysis, because such cases could have stemmed from strong genetic predisposition and their inclusion in the analysis might slightly undermine the pragmatic implications of the findings (*n* = 31).

Of the 4250 students from 24 schools who were eligible to take part in the study, 424 participants (11.24%) were absent, 55 (1.46%) refused to participate, and finally a total of 3771 students (88.73%) were examined.

### 2.2. Data Collection and Measurements

A questionnaire comprising two parts was designed for the data collection. The first part contained questions about socio-demographic details, such as age, sex, grade, region of habitation (urban/rural), starting reading age, school levels (key/ordinary), hierarchy (primary, junior and senior high school), self-assessed academic records (categories: poor, fair, average, and high), monthly household income (categories: <¥1,000 (Chinese Yuan), ¥1,000–¥2,999, ¥3,000–¥4,999, and ≥¥5,000). The second part asked about the time spent on daily activities, such as studying outside school, participating in physical activities, doing homework (<1 h or ≥1 h), and sleeping (unknown, <8 h, 8–10 h, or ≥11 h). We also probed whether students had any homework during recess and weekend tutoring and whether they controlled their time of viewing television and using computers. As for very young children who could not read or understand the questions well (such as 1–2 grade students), parents explained the questionnaire and helped to choose the appropriate answers.

An eye examination for each participant followed the filling-out of the questionnaire. The exam was performed by a team formed by an ophthalmic nurse and an ophthalmologist. VA was measured using the Standard Logarithmic Visual Acuity E Chart at 5 m. VA was measured both with and without glasses. The staff started the test at line 0 logMAR (20/20). If the student could identify the optotype, the doctor pointed to the next smaller line. If he/she failed, the doctor pointed to the next bigger line. The time was limited to 5 s/response. If the student took a longer time to identify an optotype, the staff would shift to another optotype. No participant was allowed to strain their eyes to gain better acuity during the examination. Students with uncorrected VA of 0.30 logMAR (Snellen, 6/12 or 20/40 or less) or more in either eye were regarded as having VI. VI included low vision and blindness. Low vision was defined as having VA greater than 20/200 but less than 20/60. A VA less than 20/200 (logMAR ≥ 1.00) in either eye was defined as blindness [29].

Weight was measured in kilograms and height in centimeters using a wall-mounted measuring tape. The participants were told to remove their shoes and any heavy objects from their bodies before the measurements. BMI was calculated directly from weight in kilograms and height in meters (kg/m^2^). BMI was categorized into underweight (<18.5 kg/m^2^), normal weight (<25 kg/m^2^), overweight (<30 kg/m^2^), and obese (≥30 kg/m^2^) [30]. Overweight and obese categories were combined into overweight/obese because of the small number of respondents (*n* = 58).

### 2.3. Quality Control

The questionnaire was designed by four experts (an ophthalmologist, an epidemiologist, a nutritionist and a health care teacher) with over 15 years of working experience. We delivered 100 questionnaires as a preliminary survey to test whether the questions were understood clearly by the students and modified the questionnaire according to students’ feedback. Then, the questionnaire was presented to seven experts in areas of Wuhan University such as epidemiology, ophthalmology, and education. These specialists accredited the face validity and authenticity of the questionnaire before our formal use. The project was administered by a number of graduate students of the School of Public Health of Wuhan University and the faculty of Huangpi District People’s Hospital. The explanations were standardized and all the research team members were trained before administering the survey. Data collected with questionnaires and eye-examinations were double-entered into a database created with Epidata by two independent staff members. All data were checked for consistency between by the two members.

### 2.4. Data Analysis

Chi-square tests or analyses of variance, depending on the variables, were used to analyze the participants’ characteristics stratified by BMI categories. Logistic regression modeling was run to analyze the association of BMI and VI, adjusted respectively for (1) age and gender; and (2) age, sex, level, district, hierarchy, academic performance, starting reading age, monthly family income, homework during recess, hours of daily outdoor activities, time of daily homework, weekend tutoring, daily sleep hours, and control of TV and computer use. Odds ratios (OR) were calculated as estimates of the correlations. All *p* values were 2-sided and *p* < 0.05 was considered statistically significant. Most of statistical analyses were carried out with SPSS 22.0 (IBM-SPSS, Chicago, IL, USA). Threshold effect analysis and dose–response analysis with curve fitting were conducted using Empower (R) (www.empowerstats.com, X & Y solutions Inc., Boston, MA, USA) [31].

### 2.5. Ethical Issues

The study was authorized by the Ethics Review Committee of Wuhan University (No. 201606), according to the principles of the Declaration of Helsinki. All participants voluntarily took part in the study and submitted their written consent to the researchers.

## 3. Results

Table 1 shows the characteristics of the sample by BMI categories. There were 729 students with VI in at least one eye, accounting for 19.33% of the total participants (3771). Tests of the group differences through multivariate analyses of variances revealed the following: compared with underweight participants, normal weight and overweight/obese participants were more likely to be older males, live in urban areas, have average to high grades, and attend key schools, be in high schools, but participate in less outdoor activities. Compared with underweight and normal weight participants, overweight/obese ones were more likely to do homework during recess and do it for more than 1 h each day (*p* < 0.001).

Table 2 shows the association between BMI and VI from the logistic regression analysis. The prevalence of VI in the overweight/obese group (64.20%) was significantly higher than the underweight and normal groups (17.80% and 10.50%, respectively; *p* < 0.001). Compared to normal weight and underweight, overweight/obese showed a close connection with VI in the age/sex-adjusted model (OR = 16.16, 95% Confidence Interval (CI): 12.37–21.09). In addition, there was still a close linkage of overweight/obese to VI after adjusting for other factors affecting vision in a multivariate model (OR = 8.32, 95% CI: 6.13–11.30).

**Figure 1:**
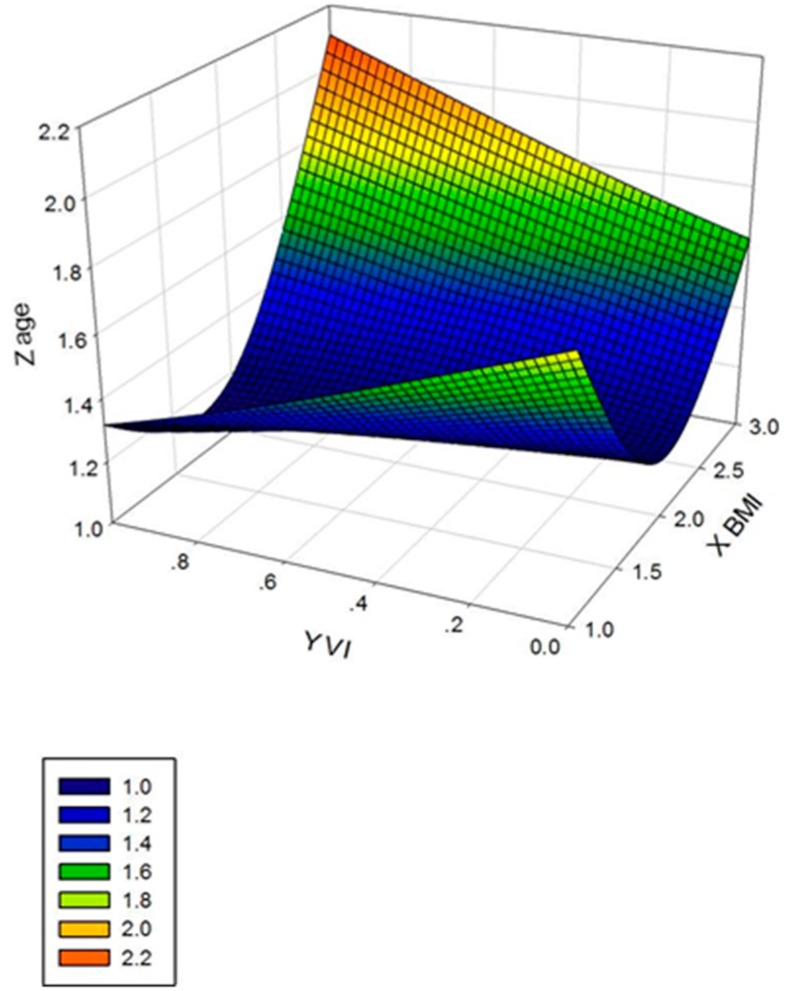
The Relationship of Boday Mass Index (BMI), Visual Impairment (VI) and Age based on the 3D Image. Age was divided into three grades according to the primary school (6–13 years), junior high school (14–16 years) and senior high school (≥17 years). BMI was categorized into underweight, normal, overweight/obese.

In analyses stratified by hierarchy (Table 3), the prevalence of VI significantly increased as BMI increased in primary, junior and senior high schools (*p* < 0.001). Similar to the whole population, the inverse association of overweight/obesity with VI was consistently present in all three hierarchies. The relationship of BMI and VI in hierarchy is shown in Figure 1 below. Figure 1 shows that there were more students with VI as age and BMI increased. High school students had higher probability of having VI than middle school students.

**Figure 2:**
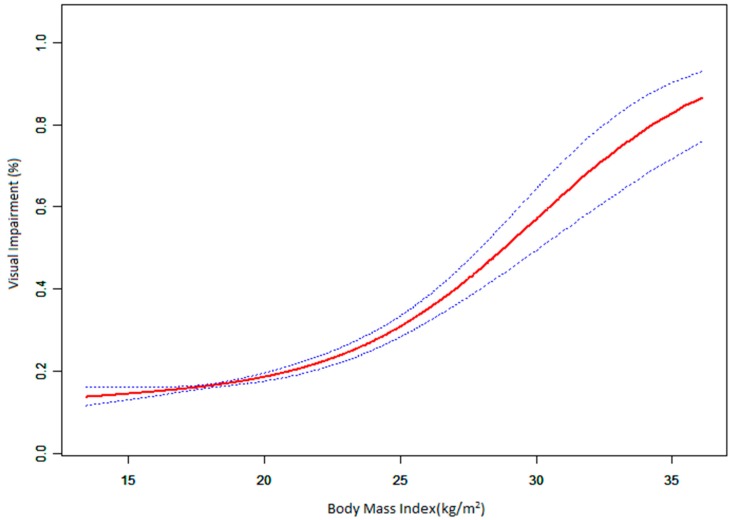
The dose–response relationship of BMI and VI. Adjusted for age, sex, level, district, hierarchy, scores, starting reading age, monthly family income, homework in recess, hours of daily outdoor activities, time of daily homework, weekend tutoring, daily sleep hours, control of TV and computer use.

The relationship between BMI and VI appeared to be nonlinear (Table 4 and Figure 2). The risk of VI increased with BMI rising to the turning point (BMI = 19.81 kg/m^2^). With a BMI < 19.81 kg/m^2^ as the threshold, the estimated dose–response curve was nearly a horizontal line. However, when the BMI was above 19.81 kg/m^2^, the prevalence of VI increased with increasing BMI and the correlation coefficient (β) was 1.20 (95% CI: 1.15–1.25), implying that the risk of VI incidence could increase by 20% for each increase 1 kg/m^2^ when the BMI was above 19.81 kg/m^2^.

## 4. Discussion

This study found the prevalence of VI among students in Central China and revealed a nonlinear association between three levels of BMI and VI. Overweight/obese was associated with increased risk of VI after adjustment for possible confounding factors. We discuss the results separately below and extend to some practical implications.

The prevalence of VI in our study (19.33%) was higher than that in migrant children aged 7–12 in the suburbs of Shanghai (13.33%) [32], in urban children aged 5–15 of Chile (15.8%) [33], in students aged 5–15 in rural areas of Beijing (8.18%) [24], in students aged 6–15 in a suburb of Chongqing (7.69%) [34], and in an urban Indian sample aged 5–15 (6.4%) [35]. However, it was lower than that reported in an urban sample of children aged 5–15 from Guangzhou (22.30%) [26]. These differences may be due to the fact that the subjects of our research contained high school students, whose VI rate is higher than other age groups, as was shown in our study. Alternatively, it could be due to different economic status, academic pressure, and/or access to eye care services.

Our study showed that there was a nonlinear dose–response relationship between BMI and VI. Two studies found that the prevalence of overweight/obesity was higher among those children with VI problems than in normal ones [22,36]. Another study found a significant positive correlation between low vision and BMI [19]. The mechanism underlying the relationship might be that VI in the students was caused by overweight or obesity. The risk of becoming obese in people with VI was over 1.5 times higher than that in the general population [21]. It was possible that those with VI rarely engaged in sports activities, spent more time in front of the screen, and therefore had higher BMIs [37,38]. In recent years in China, students have spent more hours in front of TV and computer screens because of the rapid economic development and advances in global technology [27]. However, they also spend less time than recommended on physical activities [39,40,41,42]. TV viewing is often accompanied with snacks or food and less outdoor activities, therefore tending to be related to lower vision and higher BMIs [43,44]. Our study showed that students with VI had lower levels of physical activity than children with normal vision, which was in line with other studies [40,41,45,46]. Higher levels of overweight or obesity combined with less time in physical activities may be the underlying causes of a greater risk of VI. Therefore, school students should be encouraged to do more outdoor activities and spend less time on TV and computers [47].

Obesity in school-age students is currently a major public health concern [48]. Since overweight/obesity (BMI ≥ 25) is an independent risk factor of VI, it is necessary to control the overweight/obesity. As is commonly known, physical exercise is critical to control the overweight/obesity of childhood and adolescence [49,50]. The CDC had also suggested that comprehensive schools should encourage physical activity among students [51]. Accordingly, we should take the necessary measures such as specific education to encourage students to have outdoor activities for at least one hour per day, and go outside for outdoor activities during class recess, so as to reduce trends in BMI. The situation at the time of the research was that there was only one trained health care teacher responsible for the students’ health care for an entire school. It would outstrip her/his service capacity to provide additional educational support to VI children. It was worrisome that there were neither enough teachers nor enough services for students’ eye health programs. In order to provide targeted care and management for students with VI, it is necessary to improve the school healthcare provision systems and the accessibility to eye care services in China. There should also be more training for existing teachers so they could incorporate children with VI into existing programs.

There are some limitations of our study. First, given the cross-sectional design, we could not draw any firm conclusions about the cause and effect of this association. Contrary to our hypothesized causal direction, VI could have led to overweight or obesity in the students, because students with VI would feel inapt with outdoor activities and thus resort to indoor solitary ones. From a longitudinal perspective, overweight and VI could even affect one another in a vicious cycle. Thus, it is necessary to do longitudinal studies in the future to explore the bidirectional causality of VI and BMI. Second, we have not controlled for any potential factors that might explain the association between levels of BMI and VI, such as parental overweight or VI that might have indicated hereditary causes. Moreover, we have not discussed the cause and severity of the VI, and the relationship between BMI and different severity grades of VI. Still little is known about how BMI interacts with different levels of VI. Future studies may control the BMI of school-age students and observe the changes of their vision, so as to determine cost-effective interventions of VI.

## 5. Conclusions

In summary, BMI is significantly associated with the prevalence of VI. In particular, our research shows that there is a nonlinear dose–response between BMI and VI, with a significant growth when BMI is more than 19.81 kg/m^2^. Because many arising vision conditions in childhood can be prevented or treated successfully with early interventions, our findings are useful for planning and implementing prevention programs for VI [52].

## Figures and Tables

**Table 1:** Baseline Characteristics of Students by Body Mass Index (BMI) (kg/m^2^) Category and Tests of Group Differences.

	Underweight	Normal	Overweight	*p* Value
	(*n* = 1733)	(*n* = 1653)	(*n* = 385)	
Age (years)	9.97 (2.52)	12.89 (3.09)	13.48 (3.00)	<0.001
Male (%)	848 (48.93)	930 (56.26)	233 (60.52)	<0.001
BMI (kg/m^2^)	16.14 (1.33)	20.86 (1.67)	27.01 (2.53)	<0.001
Urban (%)	948 (54.70)	1002 (60.62)	236 (61.30)	<0.001
Visual Impairment (%)	309 (17.80)	173 (10.50)	247 (64.20)	<0.001
Average to high (%)	1066 (61.51)	1081 (65.40)	257 (66.75)	<0.001
Key schools (%)	555 (32.03)	684 (41.37)	167 (43.38)	<0.001
High school students (%)	77 (4.44)	445 (29.92)	126 (32.73)	<0.001
Outdoor activity (h)	1.93 (1.06)	1.88 (1.02)	1.05 (0.64)	<0.001
≥1 h homework	1067 (61.70)	989 (59.83)	267 (69.35)	<0.001
>¥5,000 Monthly income (%)	619 (35.72)	550 (33.27)	144 (37.40)	0.460
Doing homework during recess (%)	481 (27.76)	434 (26.26)	109 (28.31)	0.005
Starting reading age (years)	3.64 (1.27)	3.60 (1.19)	3.57 (1.28)	0.492
Weekend tutoring (%)	417 (24.06)	366 (22.14)	101 (26.23)	0.165
<8 Sleep hours/day	538 (31.04)	508 (30.73)	127 (32.99)	0.688
Control TV and computer use (%)	958 (55.28)	915 (55.35)	221 (57.40)	0.737

Continuous variables are expressed by means (± Standard Distribution) and classification variables are expressed as a percentage.

**Table 2:** The association between BMI and Visual Impairment (VI) in the whole population.

BMI Category	Count	VI Prevalence	Non-Adjusted Model	Model I ^b^	Model II ^c^
	(VI)	(%)	(95% CI LL, UL) ^a^	(95% CI LL, UL)	(95% CI LL, UL)
Underweight	1733 (309)	17.80	1.86 (1.52, 2.27)	1.61 (1.29, 2.01)	1.95 (1.52, 2.50)
Normal	1653 (173)	10.50	1.00	1.00	1.00
Overweight/Obese	385 (247)	64.20	15.31 (11.79, 19.88)	16.16 (12.37, 21.09)	8.32 (6.13, 11.30)
*p* Value		<0.001	<0.001	<0.001	<0.001

^a^ 95% Confidence Interval (CI) lower limit (LL), upper limit (UL); ^b^ Adjusted for age and sex; ^c^ Adjusted for age, sex, level, district, hierarchy, scores, starting reading age, monthly family income, homework in recess, hours of daily outdoor activities, time of daily homework, weekend tutoring, daily sleep hours, control of TV and computer use.

**Table 3:** The association between BMI and VI stratified by hierarchy.

BMI Categories	Counts	VI Prevalence	Non-Adjusted Model	Model I ^a^	Model II ^b^
	(VI)	(%)	(95% CI LL, UL)	(95% CI LL, UL)	(95% CI LL, UL)
Primary School					
Underweight	1469 (268)	18.20	1.23 (0.98, 1.55)	1.16 (0.91, 1.47)	1.42 (1.07, 1.89)
Normal	828 (127)	15.30	1.00	1.00	1.00
Overweight/Obese	159 (74)	46.50	4.81 (3.34, 6.92)	4.88 (3.37, 7.06)	2.73 (1.79, 4.18)
*p* Value		<0.001	<0.001	<0.001	<0.001
Junior High School					
Underweight	187 (24)	12.80	1.36 (0.79, 2.36)	1.33 (0.76, 2.34)	1.64 (0.82, 3.29)
Normal	380 (37)	9.70	1.00	1.00	1.00
Overweight/Obese	100 (79)	79.00	34.87 (19.36, 62.83)	37.35 (20.32, 68.65)	22.93 (10.68, 49.25)
*p* Value		<0.001	<0.001	<0.001	<0.001
Senior High School					
Underweight	77 (17)	22.10	13.73 (5.86, 32.17)	15.39 (6.42, 36.86)	18.62 (6.86, 50.54)
Normal	445 (9)	2.00	1.00	1.00	1.00
Overweight/Obese	126 (94)	74.60	142.31 (65.73, 308.07)	159.9 (71.57, 357.26)	98.04 (39.44, 243.68)
*p* Value		<0.001	<0.001	<0.001	<0.001

^a^ Adjusted for age and sex; ^b^ Adjusted for age, sex, level, district, hierarchy, scores, starting reading age, monthly family income, homework in recess, hours of daily outdoor activities, time of daily homework, weekend tutoring, daily sleep hours, control of TV and computer use.

**Table 4:** Threshold effect analysis of BMI on VI.

BMI	Crude β/OR (95% CI)	*p* Value	Adjusted β/OR (95% CI) *	*p* Value *
<20.41	0.92 (0.88, 0.96)	0.0002		
≥20.41	1.33 (1.27, 1.38)	<0.0001		
<19.81			0.86 (0.80, 0.92)	<0.0001
≥19.81			1.20 (1.15, 1.25)	<0.0001

* Adjusted for age, sex, level, district, hierarchy, scores, starting reading age, monthly family income, homework in recess, hours of daily outdoor activities, time of daily homework, weekend tutoring, daily sleep hours, control of TV and computer use; β: correlation coefficient; OR: odds ratio.
